# Modular tapered conical revision stem in hip revision surgery: mid- term results

**DOI:** 10.1186/s12891-020-03886-y

**Published:** 2021-01-06

**Authors:** Loris Perticarini, Stefano Marco Paolo Rossi, Alberto Fioruzzi, Eugenio Jannelli, Mario Mosconi, Francesco Benazzo

**Affiliations:** 1 Sezione di Chirurgia Protesica ad Indirizzo Robotico - Unità di Traumatologia dello Sport, U.O. Ortopedia e Traumatologia Fondazione Poliambulanza, Via Bissolati 57, 25124 Brescia, Italy; 2 Dipartimento dell’Anca e Traumatologico, IRCCS Policlinico San Donato, Milan, Italy; 3 Clinica Ortopedica e Traumatologica, Fondazione IRCCS Policlinico San Matteo, P.le Golgi 19, Pavia, Italy; 4 Università degli Studi di Pavia, Pavia, Italy

**Keywords:** Conical revision stem, Femoral bone defects, Hip revision surgery, Modularity, Periprosthetic femoral fracture

## Abstract

**Background:**

The aim of this paper is to evaluate the clinical and radiological outcomes of a fluted tapered modular distal-fixation stem at medium to long-term follow-up. The hypothesis of this investigation was to verify if the use of this implant design may have provided potential advantages in femoral revisions and post-traumatic instances where the restoration of the anatomy was the prime concern.

**Methods:**

We retrospectively reviewed 62 cases of femoral revision surgeries, performed in Paprosky type IIIA and IIIB bone defects between January 2001 and December 2011 with a mean follow-up of 8.5 ± 1.5 years (range 5.1–15.9 years) where a modular fluted stem was used. The clinical assessment was performed with the Harris Hip Score (HHS), and the radiographic evaluation was carried in order to assess the stability of the femoral component. Intra-operative and postoperative complications were recorded, and the rates of complications and revisions for any cause were determined.

**Results:**

Mean HHS improved 35.4 points from the preoperative assessment. Radiographic evaluation showed a stable stem anchorage in 90.3% of the cases at the last follow-up. Five (8%) implants required additional surgery. Neither breakage of the stem nor loosening of the taper junction were recorded. Kaplan-Meier survivorship was 89.4% (CI: 88.8–90%) for any complication and 92.3% (CI: 91.8–92.7%) according to revision for any causes at 81 months follow-up.

**Conclusions:**

Our findings suggest that this stem design is a reliable option in cases of complex femoral bone defects, as well as in cases with high functional deficiencies, with promising survivorship.

## Background

An increase in the number of revision surgery is expected over the next few decades with a growing number of primary total hip joint replacements in younger and more active patients [1, 2].

Revision surgery aims to create a stable construct, protect bone and soft tissues, fill bone defects, and restore the biomechanical function of the hip. Femur reconstruction during a revision total hip arthroplasty can be the most challenging phase of the operation [3]. Bone defects may be the results of osteolysis, infection, periprosthetic fractures, stress shielding, and implant extraction. The several classifications for the femoral bone defects proposed in the literature are treatment oriented, guiding the surgeons in selecting the right method for femoral reconstruction [4].

Cemented long femoral stems are usually indicated in elderly patients who present the most severe conditions in terms of bone quality. Cementless monolithic revision stems can be used but sometimes they may have limits. Cementless modular revision stems differ from the first two as they allow independent preparation of distal and proximal bone in the femur, as well as individual adjustment of leg length, offset, and anteversion. Fluted tapered stem designs are indicated when it is necessary to achieve axial and rotational stability distally in the femur because the proximal part can’t support fully the vertical load [5].

Although several authors have reported mid-term survival rates higher than 95%, there is still a lack of studies detailing the role of vertical stem instability in the osseointegration of fluted tapered stems [6–8]. This study aims to evaluate the clinical and radiological outcomes of a fluted tapered modular stem with distal fixation at a minimum follow-up of 5.1 years. Primary hypothesis of this investigation was that this implant could show clinical outcomes and survival rates at least comparable to those presented in previous studies with cemented or uncemented monoblock or modular stems. Secondary hypothesis was that this stem is reliable in cases of periprosthetic femoral fractures, septic or aseptic stem loosening with femoral bone defects type IIIA and IIIB according to the Paprosky classification. Finally we wanted to verify the hypothesis that the use of this implant design may provide potential advantages in the cases of femoral revision and post-traumatic instances where the restoration of the hip anatomy was the prime concern.

## Methods

Between January 2001 and December 2011, 101 patients underwent a hip revision arthroplasty using a fluted tapered modular stem (Revision Stem, LimaCorporate, San Daniele del Friuli, Italy).

14 patients died before the end of the 5 years evaluation; 25 patients were not reachable for different reasons, related to distance and unavailability for changing the address. Thus, the final study group consisted of 62 patients (Table 1), with an average follow-up of 8.5 ± 1.5 years (range 5.1- -15-9 years). There were 25 males (40.3%) and 37 females (59.7%) with a mean age of 69 ± 12.27 years (range: 29–89 years) (Table 2). 38 cases (62.2%) required an associated cup revision. In 29 cases (76.3%), we used a standard acetabular cup (2 cases Blind Cup®, 16 cases Delta TT cup® and 11 cases Trilogy TM cup®). In 9 patients (23.7%) was necessary to use an acetabular revision system (5 cases Delta TT revision cup® and 3 cases SPH Revision®). The tribology used was polyethylene-ceramic in 25 cases, polyethylene-metal in 10 cases and ceramic-ceramic in 3 cases.

**Table 1:**
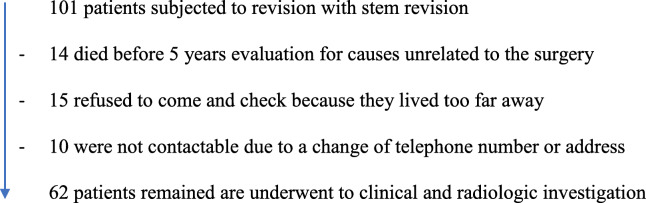
Flowchart of selected patients

**Table 2:** Demographic data, diagnosis and Paprosky bone defect classification

	Minimum	Maximum	Mean	Std. Deviation
Age (y)	29	89	69.02	12.275
Weight (kg)	30	97	68.81	14.501
Height (cm)	120	184	165.66	10.070
		N	%	
Sex	Female	37	59.7	
	Male	25	40.3	
Side	Left	29	46.8	
	Right	33	53.2	
**Diagnosis**		**N.**		**%**
Aseptic loosening		38		60.7
Septic loosening		5		8.2
Periprosthetic fractures		11		18.0
Mechanical stem failure		7		11.5
Femoral nail failure		1		1.6
**Femoral bone loss**		**Paprosky**		**%**
		IIIA		91.9
		IIIB		8.1

Indications for revision were aseptic loosening of the implant in 38 (60.7%) hips, septic loosening in 5 (8.2%), periprosthetic fracture in 11 (18.0%), mechanical failure (implant breakage) of the component in 7 (11.5%), and failure of internal fixation of proximal femur fracture in 1 case (1.6%). The femoral bone loss in septic and aseptic loosening cases was classified according to the Paprosky classification as IIIA (91.9%) and IIIB (8.1%) [9].

A single senior surgeon performed all the revision procedures using a posterolateral approach. In 49 (79%) cases a transfemoral osteotomy was used to remove the previous stem, and in 6 (9.7%) of these cases a circular osteotomy was associated. The decision to use a transfemoral osteotomy in the majority of cases was dictated by the good fixation of the implant in the majority of the cases, and by the features of Revision stem that is conical and doesn’t follow the anterior bowing of the femur; the femoral canal is, therefore, easier to prepare with this approach.

The osteotomy fragment was secured after re-implantation with at least two cerclage wires in all cases. A prophylactic cerclage wire was placed in all cases of transfemoral osteotomy, and before the insertion of the stem into the femur with thin cortices in order to prevent the propagation of intraoperative cracks.

All patients were intravenously administered antibiotic prophylaxis using 1 g of Vancomycin during surgery and 500 mg every 4 h until the second day after surgery, while thromboembolism prophylaxis was performed using Enoxaparin 4000 UI/day for 30 days after surgery. All patients were allowed to stand on the second postoperative day and to progress to full weight-bearing with crutches as tolerated. Clinical and radiographic evaluations were carried out before surgery, as well as at 6 weeks, and at 3, 6 and 12 months after the surgery, and then at 1-year intervals until the final follow-up visit. Clinical assessment was comprised by a detailed medical history, a physical examination, and the Harris Hip Score [10]. Femoral component stability was determined using the criteria described by Engh et al. [11]. Subsidence of the femoral component was measured as the change in the distance from the center of the femoral head to the most proximal point on the lesser trochanter. Heterotopic ossifications were evaluated according to the Brooker classification [12].

Statistical analysis was performed using the IBM SPSS Statistics for Macintosh (Version 22.0, IBM, Armonk, NY, USA). At the minimum follow-up of 63 months, we calculated the rate of complications and of revisions for any cause using Kaplan-Meier methodology.

### Description of the implant

The tapered fluted modular Revision (Lima Corporate, San Daniele del Friuli, Italy) stem is made of titanium alloy Ti6Al4V and has a straight distal anchoring module of conical shape (with a 1° 36′ angle). The body lengths are either 140 or 200 mm, with 14–26 mm in diameter (1 mm increments), with eight longitudinally-oriented anchoring blades. A Morse taper (which is deflected by 4° from the long axis of the distal part) ensures the assembly of the distal and proximal elements of the stem; a locking screw provides an additional safety tool to guarantee a stable connection of the two parts. The proximal module is available in 7 sizes (50–110 mm long with 10 mm increments), with a neck-shaft angle of 131° (40 mm offset) and 135° (35 mm offset) and a 12/14 neck cone. The vertical stability is based on the tapered shape of the body, while longitudinal blades ensure rotational stability.

## Results

At the last follow-up, the mean Harris Hip Score was 72.1 ± 15.8 (range: 23–97), with an average improvement of 35.4 from the preoperative score, which showed a mean value of 36.7 ± 12.4 (range: 5.8–54). 33.9% of the patients (21 cases) had good or excellent results, 27.4% (17 cases) had fair results, and 38.7% (24 cases) had results under 70 points.

At a mean follow-up of 8.5 ± 1.5 years (range 5.1- -15-9 years), five (8%) patients required additional surgery. Two (3.2%) patients had a traumatic periprosthetic fracture at 6 and 8 years follow up and were treated with open reduction and internal fixation. Three (4.8%) patients underwent surgery for an acetabular cup failure due to liner wear. One (1.6%) patient had an episode of dislocation and experienced reduction under anesthesia. One (1.6%) patient had a wound infection treated with oral antibiotics. Therefore, no revision was performed in this study for a mechanical failure of the implant. Seven (11.3%) patients developed heterotopic ossifications: four (6.5%) cases were classified as Brooker II, two (3.2%) as Brooker III and one (1.6%) as Brooker IV.

**Fig. 1:**
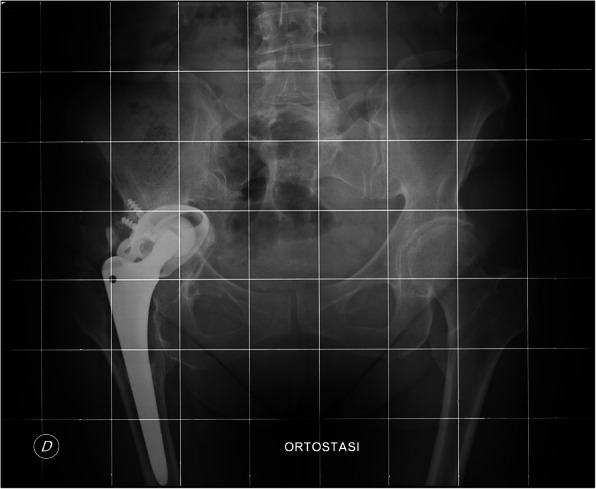
X-ray of aseptic loosening of right hip of 54 years old female

**Fig. 2:**
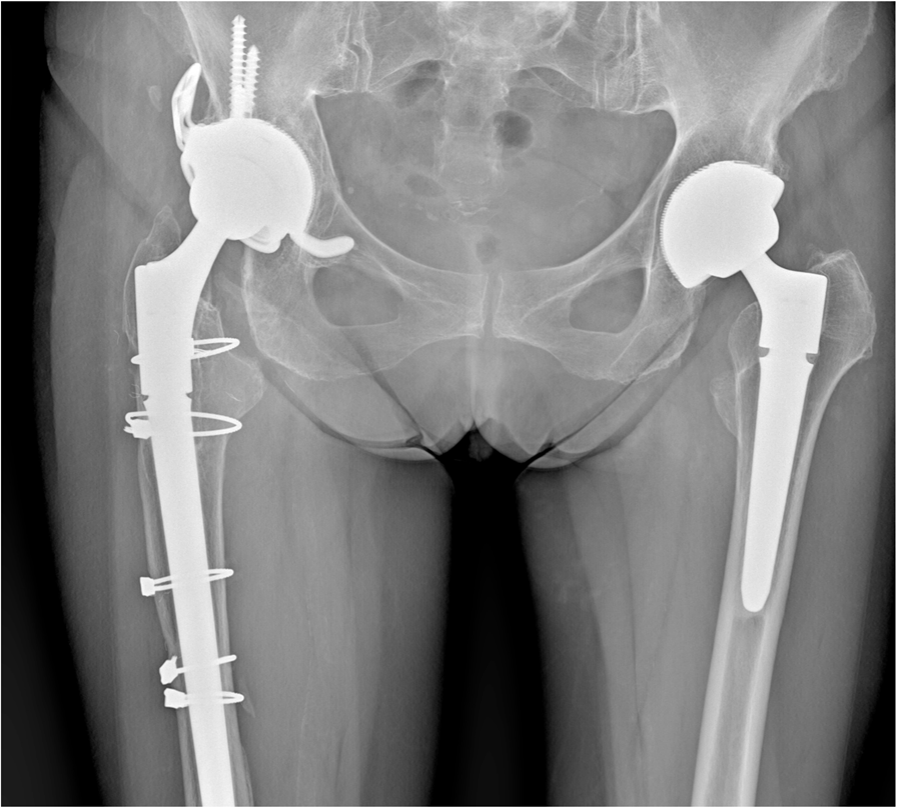
X-ray at 7 years’ follow-up after revision surgery

At the last follow-up, 56 (90.3%) cases showed radiographic evidence of a stable bone fixation (Figs. 1-2). In one (1.6%) case, the formation of a pedestal was observed, while in two (3.2%) cases, the appearance of radiolucent lines surrounding the implant interface was noted.

Early (within 3 months of surgery) subsidence (> 2 mm) of the stem occurred in 4 (6.5%) cases: one (1.6%) stem subsided by 2 mm, one (1.6%) by 3 mm, one (1.6%) by 4 mm, and one (1.6%) stem by 6 mm, with a measured mean value of 3.8 mm (range: 0–6 mm; SD: 1.7 mm). After the subsidence was first observed, none of these 4 hips showed any further signs of progression at subsequent follow-up.

### Intraoperative complications

In four (6.5%) cases, an intraoperative femoral fracture with diaphyseal split occurred, one proximal and three distal. Two of the four diaphyseal split fractures occurred during stem insertion, whereas the other two occurred during cement removal. All the diaphyseal split fractures were treated with plate and cerclage wires and subsequently healed in situ with stable bone fixation of the stem. One additional (1.6%) anterior cortical perforation occurred during stem insertion; due to patient’s comorbidity and the poor functional request it was decided to treat it conservatively. All the fractures eventually healed without any further intervention and went on to stable bone fixation of the stem. In two cases (3.2%), there was a leg length discrepancy less than 1.5 cm that proved not correctable with the revision procedure.

### Survival analysis

**Fig. 3:**
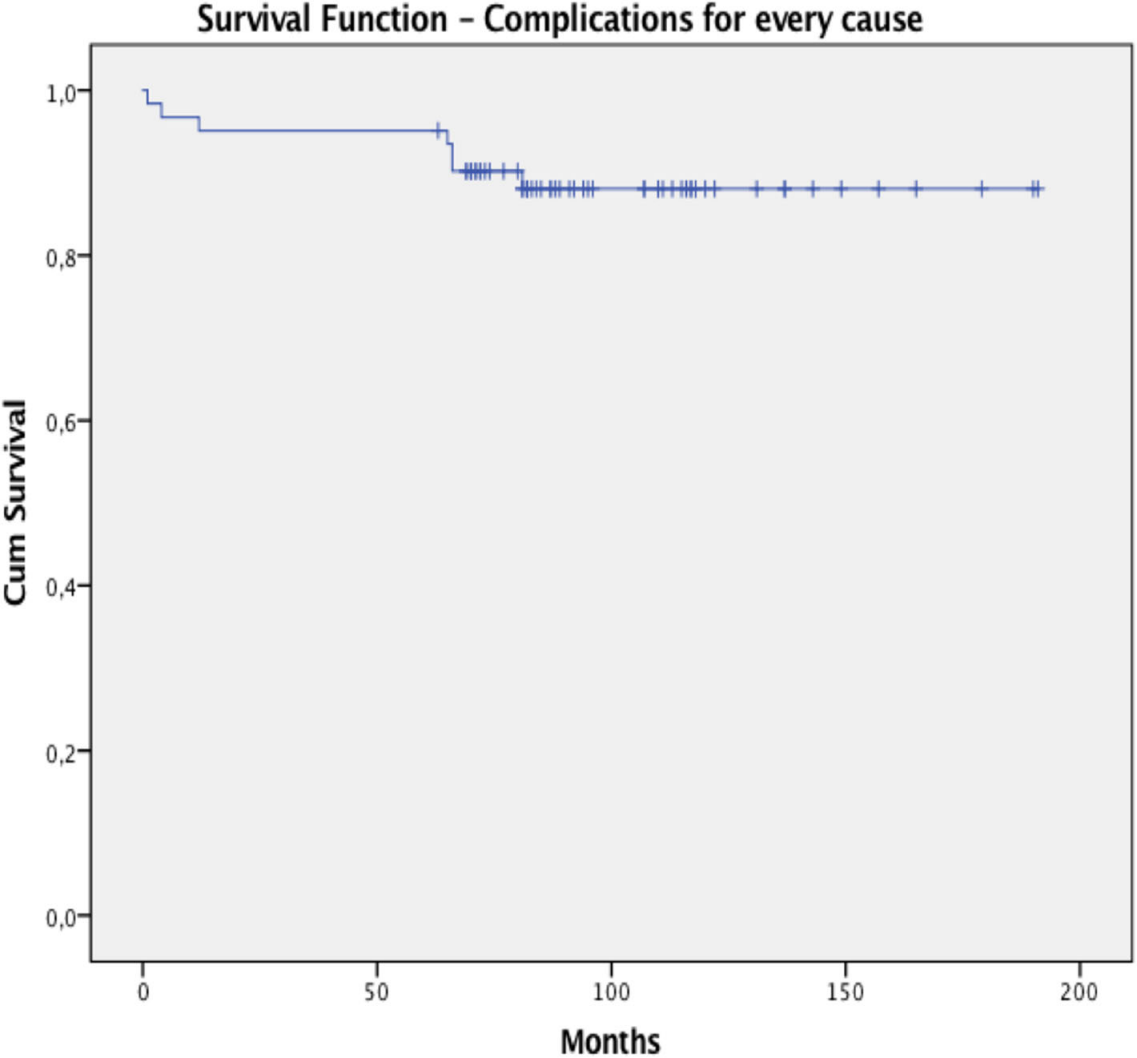
Kaplan-Meier survival rate for complications for every cause

**Fig. 4:**
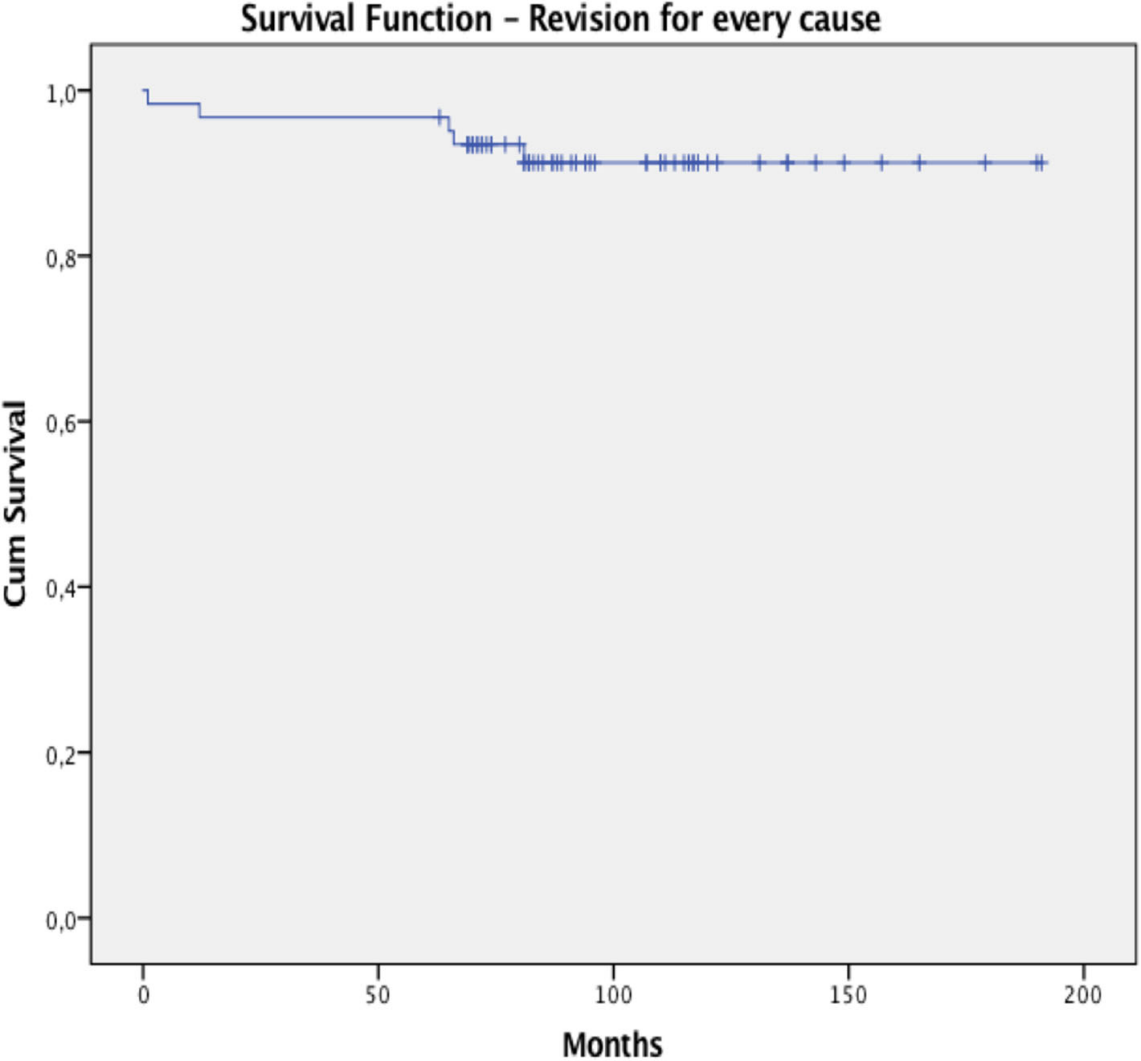
Kaplan-Meier survival rate for revision for every cause

Kaplan-Meier Survivorships has been assessed for the final study group of 62 patients reaching a maximum period of 15.9 years, and we decided to exclude the patients died with the implant in situ. Considering complications for any cause as the endpoint, the survival rate was 89.4% (CI: 88.8–90%) at 6.75 years. When taking as an endpoint revision for every cause, the survival rate was 92.3% (CI: 91,8% - 92,7%) at 6.75 years, as shown in Table 3. The survival curve for both analyses reached the lowest rate at 6.75 years, as showed in Figs. 3 and 4.

**Table 3:** Survivorships for complications and revision for every cause

			Statistic	Std. Error
Survivorship for Complications for every cause	Mean		0.89450	0.003143
	95% Confidence Interval for Mean	Lower Bound	0.88822	
		Upper Bound	0.90078	
Survivorship for Revision for every cause	Mean		0.92337	0.002225
	95% Confidence Interval for Mean	Lower Bound	0.91892	
		Upper Bound	0.92782

## Discussion

The conical stems with fins conceived by Wagner to address femurs with a failed prosthetic stem, work bypassing the proximal portion of the femur (usually affected by variable bone loss, deformity or fracture). The load is transmitted to the femoral diaphysis, with an initial mechanical fixation both axially and rotationally. A secondary osteointegration occurs, and the design has shown to provide early repair of proximal osteotomies and pre-existing fractures.

Modularity of the proximal portion has been added to satisfy the unmet needs coming from the second generation of Wagner type stems (monoblock). The concept of the stem adopted in this series is based upon the possibility of fitting the stem as distal as needed, in the portion of bone where fixation is optimal, and to reconstruct the proximal portion with the length of body needed, form 50 mm to 110 mm. The potential advantage of using modular distal-fixation stems is that the modularity facilitates the intraoperative adjustment of the leg length, femoral offset, and neck version [13]. This main feature is coupled with the possibility of optimizing the version of the neck, and therefore theoretically reducing the incidence of dislocation.

In the existing literature, there are conflicting opinions regarding the clinical efficacy of stem modularity in revision THA. Regis et al. [14] analyzed the rate of dislocations in two groups of patients undergoing THA revision with standard-modularity stem and an increased-modularity stem; the authors reported that dislocations were observed early in both groups, and concluded that the use of an increased-modularity revision stem alone did not prove to be effective in reducing the risk of postoperative dislocation. A retrospective analysis of 145 hips undergoing revision, showed at 2 years follow-up no statistically significant difference in complication rates (intraoperative fracture, dislocation and aseptic loosening), functional outcomes, and radiographic parameters both in modular and monoblock splined, tapered titanium stems [15]. However, this study presents a limitation, consisting in a limited number of patients with severe degree of femoral bone loss (grade IIIB and IV) treated with femoral monoblock stem, and therefore the two groups are not fully homogeneous. Regis et al. [16] in their retrospective study of 68 consecutive hip undergoing femoral revision using a cementless monoblock stem, reported seven intraoperative fractures of greater trochanter; furthermore, eight stems (19.5%) showed subsidence ≥10 mm and four dislocations within the first 24 days of surgery (9.7%). On the other hand, the short-term results using tapered fluted modular distal-fixation stems have been good, with mechanical failure rates ranging from 1.4 to 4.3% at 2.3 to 4.0 years of follow-up [17, 18]. In the study by Park et al. [19] no femoral re-revision was performed because of mechanical failure of the stem in the 62 hips at 4.2 years of follow-up. The mean stem subsidence was 1.1 mm, and the complications included intraoperative diaphyseal split fractures (6%), cortical perforations (6%), and dislocations (5%). We have indeed obtained a satisfactory result from this point of view, given the small number of stems that have undergone a distal migration and only one case of dislocation. All cases showing a distal migration were treated with the first version of Revision stem, where the diameter was set to steps of 2 mm, and therefore anchorage and fitting was in these cases suboptimal. Our results are comparable with those of previous studies, reporting on considerable improvements in component fixation with no increase in the complication rates using a modular fluted and tapered grit-blasted stems [20]. It should be noted that the 1.6% rate of dislocations in this study is lower than recently reported dislocation rates ranging from 5% using the same stem [19] to 7.4% [21] or 8.4% [22] in large cohorts of revision THAs with monoblock stems. We believe that the possibility to optimize the biomechanical parameters may have contributed to the lower rate of dislocations in our series.

However, the modularity exposes to the risk of mechanical failure of the taper at the neck-stem junction; a high BMI, a high level of functional demand, a narrow medullary canal, and poor proximal bone support are factors that should be taken carefully under consideration when using a modular stem as they may influence negatively the outcomes of the revision procedure. Furthermore, one critical risk factor for the mechanical failure of the implant is fretting, which is defined as the damaging mechanical action that occurs when the contact components are subjected to cyclic loading, resulting in an oscillatory micro-movement [23]. It is, therefore, essential to ensure that there is a perfect placement of the Morse taper junction during the surgical procedure; the Revision stem used in our study provides a Morse taper to ensure the assembly of the distal and proximal elements, and a locking screw, as an additional safety tool for guaranteeing a stable connection of the two parts. Garbuz et al. [24] showed one stem fracture at the modular junction of 31 femoral revisions with a modular distal-fixation fluted tapered stem. This complication was also reported in association with monoblock stem designs intended for distal fixation [25]. In the patients included in our study, there was no mechanical failure of the femoral component; in particular, we did not report any complications in the stem-neck junction, confirming the excellent resistance of the implant. We should also consider that patients undergoing revision surgery for femoral issues, are generally less demanding in terms of physical activity, and consequently the classical factors against modularity, such as high BMI, and/or large offset, play a minor role. The Kaplan-Meier survival analysis showed a good performance of the implant, with 88.1% survivorship after 81 months with complications for any reason as the endpoint, and 91.3% at 81 months with revision for any cause as the endpoint.

The average HHS of 71.9 points is a direct consequence of our population age and comorbidities. By the way, this finding is in line with those reported by Wirtz et al. [26], who published an improved HHS from a mean preoperative score of 37 points (range: 4–97; SD: 24) to 79 points (range: 4–100; SD: 19) at the last follow-up. We have noted a substantial increase of 35.4 points in the score from the preoperative to the postoperative time.

### Limitations

This study presents several limitations: The limitation of this study consists mainly on the retrospective design, limiting its scientific value. A second limitation is a high number of patients lost in follow up, which.

On the other hand, the validity is based on the long follow-up, showing with the final score reached, that this surgical solution is reliable.

## Conclusions

The clinical results and mechanical stability obtained with this revision stem system are comparable with those observed with other designs of cementless revision stem. Our findings suggest that this stem is reliable in cases of periprosthetic femoral fractures, septic or aseptic stem loosening with femoral bone defects type IIIA and IIIB according to the Paprosky classification.

## Data Availability

The datasets used and/or analysed during the current study are available from the corresponding author on reasonable request.

## Abbreviations

THA: Total Hip Arthroplasty
HHS: Harris Hip Score
